# Third Case of *Streptococcus suis* Infection in Greece

**DOI:** 10.1155/2015/505834

**Published:** 2015-09-08

**Authors:** Marianneta Chatzopoulou, Ioanna Voulgaridou, Dimitrios Papalas, Petros Vasiliou, Maria Tsiakalou

**Affiliations:** Department of Clinical Microbiology, General Hospital of Larissa, 41221 Larissa, Greece

## Abstract

*Streptococcus suis* is a facultative anaerobic, Gram-positive coccus that can cause severe disease to both pigs and humans. Its zoonotic potential was first recognized in 1968 when the first human case of meningitis was reported in Denmark. Since then, over 1600 human cases have been reported worldwide, the vast majority of which originated in Southeast Asia, and, thus, *S. suis* has been fairly characterized as an emerging pathogen. Infection in humans presents most commonly as bacteremia and/or meningitis while less common clinical manifestations such as endocarditis and septic arthritis can occur. *S. suis* infection is extremely uncommon in Greece and this is the third human case to be reported. Correct identification is of importance for optimization of antimicrobial treatment and epidemiological monitoring.

## 1. Introduction

*Streptococcus suis* is a facultative anaerobic, Gram-positive coccus that constitutes an opportunistic pathogen for pneumonia but a primary agent of sepsis and meningitis in pigs [1]. Its zoonotic potential was first recognized in 1968 [2] when the first human cases of meningitis were reported in Denmark. Since then, over 1600 human cases [3] have been reported worldwide the vast majority of which originated in Southeast Asia and, thus,* S. suis* has been fairly characterized as an emerging pathogen. Infection in humans presents most commonly as bacteremia and/or meningitis while less common clinical manifestations such as endocarditis and purulent arthritis can occur.* S. suis* infection is extremely uncommon in Greece and this is the third human case to be reported [4, 5].

## 2. Case Presentation

**Figure 1:**
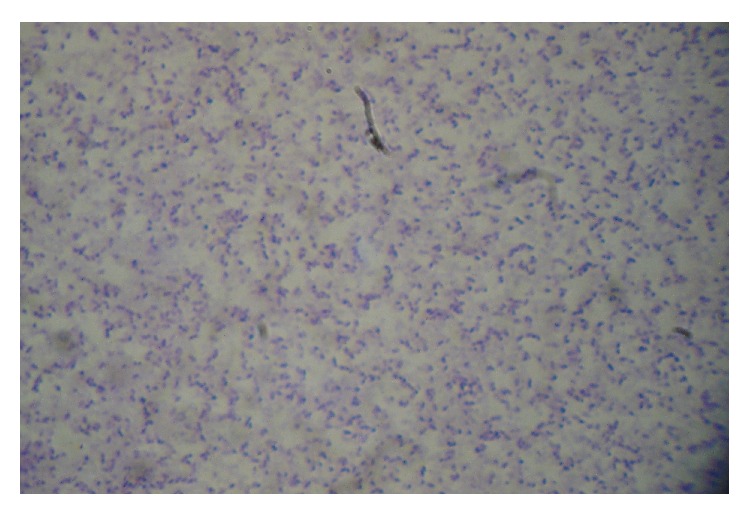
Gram stain from the CSF culture reveals ovoid-shaped Gram-positive cocci.

A 34-year-old male patient presented to the ER department with a three-day history of malaise, fever, chills, and headache not responding to paracetamol. The patient was an immigrant of Indian ethnicity working at a local piggery. He did not report any recent travels abroad, had a free medical record, and did not abuse alcohol. The patient's main clinical and laboratory findings on admission included fever (39°C), neck stiffness, partial unilateral hearing loss, leukocytosis (17400 WBCs/*μ*L), polymorphonuclear type (93,8%), normocytic anemia (Hb 11,5 gr/dL), abnormal liver function values (SGOT 54 IU/L, SGPT 62 IU/L, total bilirubin 1,62 mg/dL, and direct bilirubin 0,72 mg/dL), and increased inflammation markers (fibrinogen 890 mg/dL, CRP 30,7 mg/dL, ferritin 879,3 ng/mL, erythrocyte sedimentation rate 115 mm, and PCT 2,58 ng/mL). There were no evident abnormal signs from the cardiovascular and pulmonary systems. Brain imaging that followed showed no abnormal findings. Thereafter, a lumbar puncture was performed and urine and blood samples were collected and sent for culture. In addition, examination of a blood smear and a malaria rapid antigen test were ordered to exclude cerebral malaria. Examination of the cerebrospinal fluid revealed increased cell number (476/*μ*L) with polymorphonuclears as the predominant population (95%), low glucose levels (1 mg/dL), and high levels of protein (213 mg/dL). Gram stain of the specimen revealed the presence of Gram-positive bacteria with a somewhat coccobacillary shape (Figure 1). The clinicians were informed and empiric treatment with vancomycin and ceftriaxone was initiated while subsequent culture in the presence of optochin discs followed. After 24 hours of incubation the culture revealed small *α*-hemolytic, optochin-resistant, catalase-negative colonies growing on 5% sheep blood agar and chocolate agar. On the same day, the blood culture turned positive showing morphologically similar bacteria on Gram stain. Identification and susceptibility testing were performed with Vitek 2 Automated system.* S. suis* was identified as the responsible pathogen in both cerebrospinal fluid and blood cultures on days 3 and 4, respectively. The strain was tested susceptible to beta-lactams, macrolides, and quinolones but resistant to tetracycline (MIC > 16 *μ*g/mL). Patient's condition improved dramatically since day 3 and deescalation of treatment was decided [6] with vancomycin being discontinued on the basis of the susceptibility report. The patient was discharged a week later with persistent, unilateral, partial hearing loss and was followed up on an out-patient basis.

## 3. Discussion

*Streptococcus suis* is a zoonotic pathogen that can cause severe disease to both pigs and humans. The most common clinical manifestations in humans are bacteremia and/or septicemia, meningitis, endocarditis, purulent arthritis, endophthalmitis, and spondylodiscitis [7], while pneumonia has, also, been reported [8].* S. suis* has been characterized as an emerging pathogen. Whether this is a result of improved diagnostics or changing epidemiological characteristics is not clear [9]. The spatial distribution of disease is, however, remarkably distinct in different areas. Human infection is quite common is Southeast Asia, less common in north Europe, rare in North America, and virtually absent from Russia [10] despite the well-developed pig-rearing industry of the latter. This has been partially attributed to variations in prevalence of serotype 2 strains between different regions and continents [10] which are regarded as the most virulent. In addition, different sequence types of serotype 2 strains prevail within different geographic areas with ST1 being most prevalent in Asia and ST25 and ST28 in North America [11]. The well-recognized behavioral risk factors for infection are occupational exposure to swine, consumption of contaminated pork food, and physical contact with pigs in the presence of skin injuries [12]. Males have a mean fourfold relative risk to present with disseminated disease which, also, increases with age [12]. Some studies have shown an association with alcoholism and diabetes mellitus [7] but this is not a consistent finding [12]. Nevertheless, the exact human-pathogen interactions that lead to disseminated infection have yet to be elucidated [13] and there are studies that indicate that humans can act as asymptomatic carriers [10]. As far as we know this is the third case reported in Greece over a ten-year period since the first case report in 2005 [4, 5]. Our patient definitely belonged to a high-risk group for infection due to his occupational exposure to swine. He was of Indian ethnicity, had no underlying comorbidities, and did not report alcohol abuse.

*Streptococcus suis* is an ovoid-shaped Gram-positive coccus that forms short chains and can be easily misidentified for either* Streptococcus pneumoniae* or* Enterococcus* spp. Correct identification, which can be rather tricky especially in low prevalence settings, is of clinical importance for several reasons, the first of which is the need for optimization of antimicrobial therapy.* S. suis* strains are generally susceptible to beta-lactams [14] while enterococci show variable susceptibility to ampicillin and are considered intrinsically resistant to cephalosporins [15]. Pneumococcal resistance to beta-lactams is, also, very common [15]. On the other hand,* S. suis* strains show a high frequency of resistance to tetracyclines and macrolides [14, 16] which are frequently prescribed against pneumococcal infections [17]. In our case, the isolate was tested resistant to tetracycline but susceptible to erythromycin. Furthermore, correct identification is of public health importance.* S. suis* infection can be foodborne and while the pathogen is usually responsible for the occurrence of sporadic cases outbreaks have been reported in the literature as well [18]. It is, therefore, important to investigate and elucidate the likely source of infection. Conclusively, it is important to record the cases in order to monitor the epidemiological trends of disease in spatial and temporal terms. It is worth mentioning that* S. suis* is not a notifiable pathogen and surveillance relies only on unstructured data.

In summary of the above,* Streptococcus suis* should always be included in differential diagnosis of systemic infections even in low prevalence settings such as Greece. Clinical suspicion should be raised when the patients report a history of high-risk behavior up to two weeks prior to the onset of symptoms or when symptoms consistent with meningitis are accompanied by hearing loss [19]. Correct identification is of importance for reasons such as optimization of antimicrobial treatment and epidemiological monitoring.
